# A Streamlined Artificial Variable Free Version of Simplex Method

**DOI:** 10.1371/journal.pone.0116156

**Published:** 2015-03-13

**Authors:** Syed Inayatullah, Nasir Touheed, Muhammad Imtiaz

**Affiliations:** 1 Department of Mathematical Sciences, University of Karachi, Karachi, Pakistan; 2 Department of Mathematical Sciences, Institute of Business Administration, Karachi, Pakistan; Tianjin University of Technology, CHINA

## Abstract

This paper proposes a streamlined form of simplex method which provides some great benefits over traditional simplex method. For instance, it does not need any kind of artificial variables or artificial constraints; it could start with any feasible or infeasible basis of an LP. This method follows the same pivoting sequence as of simplex phase 1 without showing any explicit description of artificial variables which also makes it space efficient. Later in this paper, a dual version of the new method has also been presented which provides a way to easily implement the phase 1 of traditional dual simplex method. For a problem having an initial basis which is both primal and dual infeasible, our methods provide full freedom to the user, that whether to start with primal artificial free version or dual artificial free version without making any reformulation to the LP structure. Last but not the least, it provides a teaching aid for the teachers who want to teach feasibility achievement as a separate topic before teaching optimality achievement.

## Introduction

Linear programming has been an indispensable area in the progress of the computational sciences [1]. Since 1947, after World War II, linear programming gradually arose and with the passage of time has become popular among the people of various fields. It is by far the most widely used optimization model. Its impact on economic and government modeling is incredible. Today it has become the center of attention of many Mathematicians, Economists and Decision Scientists etc. Linear programming is the optimization of an outcome based on some set of linear constraints using a linear mathematical model. The origin of developing algorithms to solve a given system of linear inequalities goes back to the 19^th^ century, where they were first studied by Fourier [2]. Later, several mathematicians such as Dines and Motzkin rediscovered these algorithms [3][4].

During the course of Second World War, Dantzig formulated the largest coefficient simplex method for solving a given linear program which he presented in a conference in 1951 [5]. Later, it was also published in his famous book “Linear programming and extensions”[6]. Since then, it has become the preferred method of LP practitioners because of its efficiency [7]. Hence, it is now the most useful tool to teach and solve practical linear programming problems.

Considering its vast applicability in various fields, teaching LPs have become an important part of undergraduate and graduate courses. Because of this, many researchers are now focused in designing algorithms which are more efficient and easily implementable for classroom teaching.

Originally the simplex method developed by Dantzig was confined only to LPs having a known feasible solution, commonly referred as the initial basic feasible solution. For the LPs having no initial basic feasible solution, almost all the current variants of simplex method are applicable in two phases [8][9], called phase 1 and phase 2. In phase 1, we create a basic feasible solution by adding some (non-negative) artificial variables to the problem with an additional objective (auxiliary objective), equal to minimization of sum of all the artificial variables, called phase 1 objective. The purpose of phase 1 process is to maintain feasibility and minimize the sum of artificial variables (or in a simpler sense minimize the sum of infeasibilities) as much as possible. If phase 1 ends with an objective value equal to zero, it implies that all artificial variables have attained a value zero (means all infeasibilities have been removed) and our current basis is feasible to the original problem, then we return to the original objective and proceed with simplex phase 2. Otherwise, we conclude that the problem has no solution.

For larger LPs, implementation of traditional two phase simplex method significantly increases the number of variables, number of iterations and thus the complexity as well. From the point of view of class room teaching, it often becomes a tedious job.

Since simplex method is so far still practically the best known pivot algorithm for solving LPs [10], therefore this has arose the need of developing more general linear program solving methods in which one may start from an arbitrary initial basic solution (not necessarily a feasible one).

Arsham [11][12][13][27] presented an artificial-free algorithm named push and pull algorithm which initiates with an incomplete basic variable set (BVS). As the algorithm proceeds the variables are brought in to the basis. The Push Phase continues until the basic variable set is complete. This phase may terminate yielding an infeasible BVS. The problem then proceeds by starting the Pull Phase, which pulls the solution back to feasibility by incorporating pivot rules similar to the dual simplex method. Arsham has claimed that the push and pull algorithm is artificial-free, however, his claim would be correct if we are only concerned with artificial variables, but actually his method requires adding artificial constraints so it is not truly an artificial free method.

Papparrizos[14] presented another artificial variable free method but his method also uses additional artificial constraint with a big-M number. The method also requires a tedious evaluation of series of additional objective functions besides the original objective function. Moreover at each iteration this algorithm must check both primal and dual feasibility[12].

Arsham[15][16] proposed another algorithm for general LP models in which he claimed that his algorithm will either provide a feasible solution or will declare infeasibility after a finite number of iterations. Enge and Huhn [17] presented a counter example in which Arsham’s algorithm is declaring a feasible problem inconsistent. Here we are presenting another consistent problem which Arsham’s method declares inconsistent.

$$\begin{array}{cc} \text{Maximize} & z=3x+5y\\ \text{subject to} & \\ & \begin{array}{l} \;x\leq 4\\ \;x\leq 6\\ 3x+2y\geq 18\\ \;x\geq 0,y\geq 0 \end{array} \end{array}$$

Some other artificial free algorithms were presented [18][19][20][21][22], but all these methods are based on minimum angle approaches which differ from the simplex method.

Hence, in all Dantzig’s simplex method is better than its successor variants in terms of efficiency and as an elementary text book material as well. But from a teacher’s point of view difficulty in simplex method is that one could not learn simplex method for feasibility (simplex phase 1), before learning simplex method for optimality (simplex phase 2). So, usually students learn “*phase 2*” before “*phase 1*”, of course it sounds odd for both teachers and students.

Here in this paper, instead of developing a new method for general linear programs we are going to present a better version of simplex method which would not only hide the unnecessary elements of simplex table but also save many computations.

Furthermore Dual version of the simplex method is the most effective and efficient way to achieve the optimality of dual feasible problem [23]. So in this paper we have also presented a dual version of our method which is indeed an artificial constraint free version of the dual simplex method.

## Advantages of the New Approach

There are several advantages of the new method. For instance, it could start with any feasible or infeasible basis of an LP. In fact it could also be very useful for solving integer programming problems. This method deals with the artificial variables in an implicit manner therefore, as long as a variable is infeasible its corresponding slack variable would be invisible but implicitly after leaving the basis it would be replaced by the corresponding invisible slack variable. In this method the artificial variables are virtually present but their presence is not revealed to the user in the form of extra columns in the simplex table. This method follows the same pivoting sequence as of simplex phase 1 without showing any explicit description of artificial variables which also makes it space efficient. Due to this reason we have named our method ‘A streamlined artificial variable free version of simplex method’. Last but not the least, it is a fruitful tool for the teachers who want to teach feasibility achievement as a separate topic before teaching optimality achievement, because working rule of this method can easily be demonstrated to the students having either a little or even no prior knowledge of simplex method for optimality (phase 2). So this method would change the learning sequence by excluding the need to teach phase 2 before phase 1.

Moreover as mentioned above dual simplex method is very efficient and effective for solving a dual problem but in case of a dual infeasible problem dual simplex method requires addition of artificial constraints which makes things difficult and cumbersome in practice [24]. In contrast the dual version of our method does not require artificial constraints. This makes our methods more efficient and effective not only for attaining feasibility but also for attaining optimality. Our streamlined versions of simplex and dual simplex methods provide a way to easily implement the phase 1 of either simplex or dual simplex method. For a problem having an initial basis which is both primal and dual infeasible, our primal and dual versions provide full freedom to the user that is they can directly initiate with either the streamlined primal simplex or streamlined dual simplex method without making any reformulation to the LP structure.

## Some Basic Terminologies and Notations

A general LP problem could be considered as

$$\begin{array}{cc} \text{Maximize } & \text{​​​​​ }z=c^{T}x\\ \text{subject ​ to} & Ax=b\\ & x\geq 0,\text{​ }x\in {ℜ}^{n} \end{array}$$ (1)

where, **x** is the decision variable vector, $A\in {ℜ}^{m\times n},\;b\in {ℜ}^{m},c\in {ℜ}^{n}$ and *m* ≤ *n*

For a given basis *B* such that ***A***_*B*_ is invertible, and non-basis *N* := {1,⋯,*n*}\*B* we get

ABxB+ANxN=b⇒xB=AB-1b-AB-1ANxN (2)

By setting **x**_*N*_ = 0, a solution $x_{B}=A_{B}^{-1}b$ is obtainable. Such vector **x** is called basic solution of system (2) or system (1) for basis *B*.

Now by substituting the value of **x**_*B*_ from equation (2), the objective of system (1) can be reformulated as

$$\begin{array}{cc} \text{Maximize} & \;z+(-c_{N}^{T}+c_{B}^{T}A_{B}^{-1}A_{N})x_{N}=c_{B}^{T}A_{B}^{-1}b\\ \text{subject to} & \\ & \begin{array}{l} x_{B}+A_{B}^{-1}A_{N}x_{N}=A_{B}^{-1}b\\ x_{B}\geq 0,x_{N}\geq 0 \end{array} \end{array}$$ (3)

The problem data could be arranged in the following table, denoted by *D*(*B*), known as short simplex table [25]

$$\left[\begin{array}{ccc} c_{B}^{T}A_{B}^{-1}b & & -c_{N}^{T}+c_{B}^{T}A_{B}^{-1}A_{N}\\ A_{B}^{-1}b & & A_{B}^{-1}A_{N} \end{array}\right]$$

The *dual* of system (3) could also be written in the following form,

$$\begin{array}{cc} \text{Minimize} & y-{\left(A_{B}^{-1}b\right)}^{T}y_{B}=-c_{B}^{T}A_{B}^{-1}b\\ \text{subject to} & \\ & \begin{array}{l} (A_{B}^{-1}A_{N})Ty_{B}-y_{N}={\left(c_{N}^{T}-c_{B}^{T}A_{B}^{-1}A_{N}\right)}^{T}\\ y_{B}\geq 0,y_{N}\geq 0 \end{array} \end{array}$$ (4)

Here dual basic variables are **y**_*N*_ and non-basic variables are **y**_*B*_.

For a given basis *B*, the problem data could be read from the short table as

The current primal/dual objective value is: $z=-y=c_{B}^{T}A_{B}^{-1}b$

The primal solution is: **x**(*B*) = (**x**_*B*_, **x**_*N*_) where **x**_*N*_ = 0, $x_{B}=A_{B}^{-1}b$

The dual solution is: **y**(*B*) = (**y**_*B*_, **y**_*N*_) where **y**_*B*_ = 0, $y_{N}={\left(-c_{N}^{T}+c_{B}^{T}A_{B}^{-1}A_{N}\right)}^{T}$

**Fig 1:**
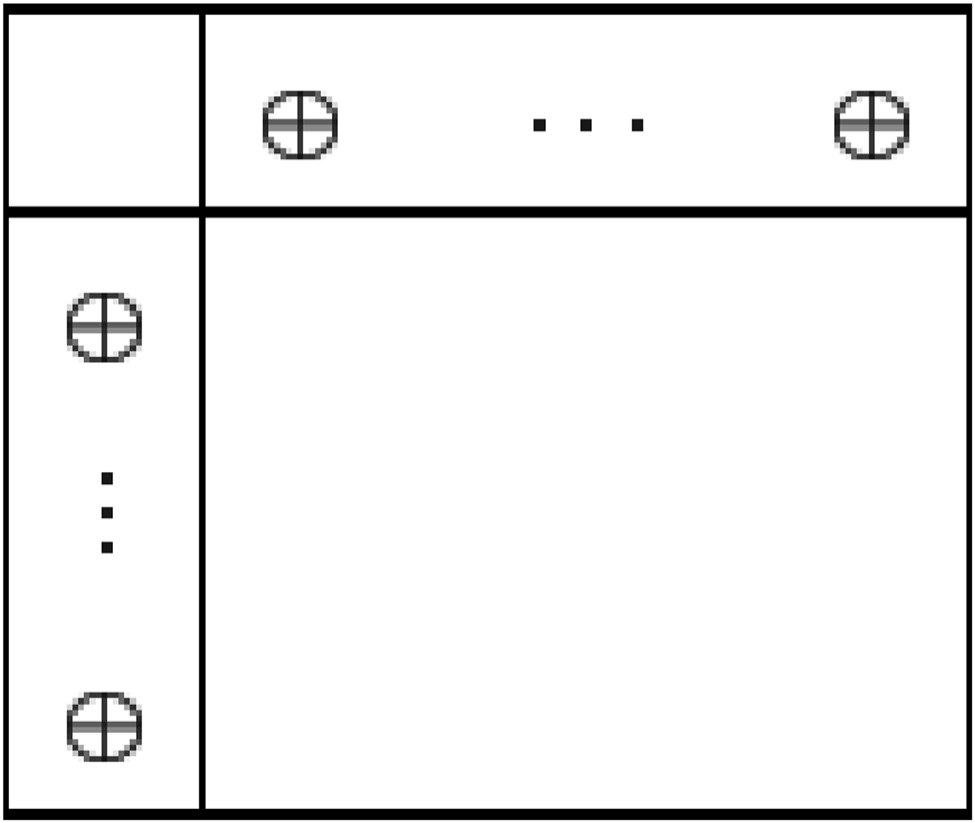
A short simplex table structure showing optimality of current basis.

A basis *B* is called *primal feasible* if **x**_*B*_ ≥ **0** or one may conclude that the primal problem has a feasible solution **x**(*B*) while if **y**_*N*_ ≥ **0** then *B* is called *dual feasible* or one may conclude that the dual problem has a feasible solution **y**(*B*). Also if *B* is both primal and dual feasible then *B* is *optimal*. Usually *d*_*B*0_ is referred as certificate column of primal feasibility; and *d*_0*N*_ is referred as certificate row of dual feasibility, see Fig. 1.

A basis *B* is called primal infeasible if there exists *i* ∈ *B* such that *d*_*i*0_ < 0 and dual infeasible if there exists *j* ∈ *N* such that *d*_0*j*_ < 0.

**Fig 2:**
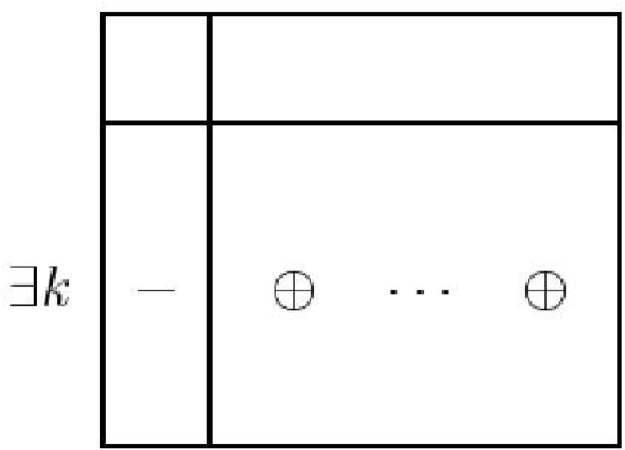
A short simplex table structure showing inconsistency of primal problem.

**Fig 3:**
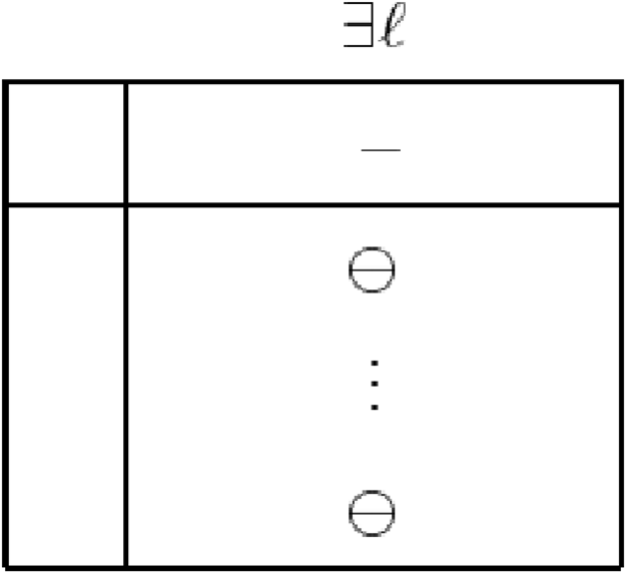
A short simplex table structure showing inconsistency of dual problem.

A short table could also be used to reveal inconsistency and unboundedness of an LP. A problem is said to be *inconsistent* if there exists a basis *B*, having a member *k*, to the problem such that *d*_*k*0_ < 0 and *d*_*kN*_ ≥ 0, see Fig. 2. A problem is said to be dual inconsistent if there exists a non-basis *N* having a member *l* such that *d*_0*l*_ < 0 and *d*_*Bl*_ ≤ 0, see Fig. 3.

Primal *unboundedness* could be deduced if a basis is primal feasible but reveals dual inconsistency; and dual problem is unbounded if a basis is dual feasible but reveals primal inconsistency.

### Pivot operation

To move through different bases of an LP one may need to apply pivot operations. A single pivot operation may be used to obtain short table of an adjacent basic solution.

For *r* ∈ *B*, *k* ∈ *N* and (*r*, *k*) being the position of the pivot element *d*_*rk*_ (≠ 0) of *D*, then one can obtain an updated equivalent short table *D*(*B*’) with a new basis *B*’ := (*B* ⋃ {*k*})\{*r*} and the new non-basis *N*’ := (*N* ⋃ {*r*})\{*k*} by performing the following operations on *D*(*B*)

d'rj:=drjdrk,       j∈N\{k}d'ik:=-dikdrk,       i∈B\{r}d'ij:=dij-drk×dikdrk,       i∈B\{r},   j∈N\{k}d'kr:=1drk

The above replacement is known as a *pivot operation* on (*r*, *k*). A *degenerate variable* is the basic variable with zero value. A pivot operation or pivot element is said to be primal (dual) *degenerate* if the corresponding primal (dual) basic variable is degenerate. A degenerate pivot is said to be *positive* (*negative*) *degenerate* if the sign of pivot element is positive (negative).

Short table before pivot (*r*, *k*)

$$\left[\begin{array}{ccc} d_{ij} & & d_{ik}\\ d_{rj} & & d_{rk} \end{array}\right]$$

Short table after pivot (*r*, *k*)

$$\left[\begin{array}{ccc} {d^{\prime }}_{ij}^{} & & {d^{\prime }}_{ir}^{}\\ {d^{\prime }}_{kj}^{} & & {d^{\prime }}_{kr}^{} \end{array}\right]$$

### Deduction of art-free auxiliary form of an LP

The term auxiliary form is commonly used in the context of simplex method as a special purpose linear program constructed by incorporating a sufficient number of artificial variables into the system in order to develop a pseudo feasible basis, and then appending an objective function of minimizing sum of all artificial variables to reach a feasible basis to the original system. Here in this section we would develop a variant of the auxiliary form of the LP, namely “the art-free form”.

Consider the following system of inequalities

$$\begin{array}{l} Ax\leq b\\ x\geq 0,\;x\in {ℜ}^{p}\\ A\in {ℜ}^{m\times p},\;b\in {ℜ}^{m} \end{array}$$ (5)

By adding the slack vector,**x**_*S*_ we can get an equivalent equality form of the above system.

$$\begin{array}{l} Ax_{O}+x_{S}=b\\ x_{O}\geq 0,\;x_{S}\geq 0\\ O:=\left\{1,2,\cdots ,p\right\},\;S:=\left\{p+1,p+2,\cdots ,p+m\right\} \end{array}$$ (6)

where **x**_*O*_ are remaining variables other than that of slack variables. Here we are allowing negative variables to reside into the basis. So, in this regard we can take *B* := *S* and *N* := *O*.

$$\begin{array}{l} Ax_{N}+x_{B}=b\\ x_{N}\geq 0,\;x_{B}\geq 0\\ x_{N}\in {ℜ}^{N},\;x_{B}\in {ℜ}^{B}\\ N:=\left\{1,2,\cdots ,p\right\},\;B:=\left\{p+1,p+2,\cdots ,p+m\right\} \end{array}$$ (7)

Here *B* may not constitute a feasible basis (because of some negative values in **b**).

We can decompose **x**_*B*_ into difference of two non-negative variables $x_{B}^{+}$ and $x_{B}^{-}$. Here the value of $x_{B}^{-}$ is showing infeasibility of the current basis *B* (Note: $x_{B}^{+}$ and $x_{B}^{-}$ work just like slack and artificial variables in usual simplex method). According to the logic of simplex method, to reach a feasible basis our aim should be to make $x_{B}^{-}$ equal to zero keeping feasibility of all other variables preserved.

$$\begin{array}{l} Ax_{N}+x_{B}^{+}-x_{B}^{-}=b\\ x_{N}\geq 0,\;x_{B}^{+}\geq 0,\;x_{B}^{-}\geq 0\\ x_{N}\in {ℜ}^{N},\;x_{B}^{+}\in {ℜ}^{B},\;x_{B}^{-}\in {ℜ}^{B}\\ N:=\left\{1,2,\cdots ,p\right\},\;B:=\left\{p+1,p+2,\cdots ,p+m\right\}\\ A\in {ℜ}^{m\times p},\;b\in {ℜ}^{m} \end{array}$$ (8)

Clearly, feasible solution set of system (8) becomes also feasible to system (7) if $x_{B}^{-}=0$. Initial set of basic feasible variables to system (8) is constructible as a sub-set from $x_{B}^{-}\cup x_{B}^{+}$, in the following paradigm,

#### Paradigm

“If $b_{i}\geq 0,\;x_{p+i}^{+}$ be the corresponding basic variable and if $b_{i}<0,\;x_{p+i}^{-}$ be the corresponding basic variable”.

Let the initial basic variable set developed by above paradigm be $x_{L}^{-}\cup x_{L^{\prime }}^{+}$, where *L* := {*p*+*i*:*b*_*i*_<0} ⊆ *B* and *L*’ := {*p*+*i*:*b*_*i*_ ≥ 0} ⊆ *B*, clearly *L*’ = *B* − *L*. Recall that non-basic variables $x_{L^{\prime }}^{-}$ would increase the extent of infeasibility if they become basic variable, so to restrict their entrance into the basis we should eliminate them from the system (8). Now, if $x_{L}^{-}$ is also empty then the basis *B* is feasible to system (7) and hence feasible to system (5), otherwise to obtain a feasible basis we would imbed an additional objective function (we may also call it as infeasibility objective function), $\text{Minimize}\sum_{p+i\in L}x_{p+i}^{-}$. Here the non-negativity $x_{L}^{-}\geq 0$ along with the new objective reinforces $x_{L}^{-}$ to zero. From system (8) one may obtain $x_{L}^{-}=Ax_{N}+x_{L}^{+}-b$, so the infeasibility objective function equivalently becomes $\text{Minimize}\sum_{p+i\in L}\left(a_{iN}x_{N}+x_{p+i}^{+}-b_{i}\right)$. The transformed system is,

$$\begin{array}{l} \text{Minimize}\sum_{p+i\in L}\left(a_{iN}x_{N}+x_{p+i}^{+}-b_{i}\right)\\ \;a_{iN}x_{N}+x_{p+i}^{+}-x_{p+i}^{-}=b_{i}\;p+i\in L\\ \;a_{iN}x_{N}+x_{p+i}^{+}\;=b_{i}\;p+i\in L^{\prime }\\ \;x_{N}\geq 0,\;x_{L}^{+}\geq 0,\;x_{L}^{-}\geq 0,x_{L^{\prime }}^{+}\geq 0\\ \;x_{N}\in {ℜ}^{N},\;x_{L}^{+}\in {ℜ}^{B},\;x_{L}^{-}\in {ℜ}^{B},x_{L^{\prime }}^{+}\in {ℜ}^{B}\\ N:=\left\{1,\cdots ,p\right\},\;B:=\left\{p+1,\cdots ,p+m\right\},\;L=\left\{p+i:b_{i}<0\right\},L^{\prime }=B-L \end{array}$$ (9)

**Note:** Throughout this paper, we are overloading the addition ‘+’ and negative ‘-’ operations for affine translations to a set Ω of real numbers by a number *a* as Ω + *a* := {*x*+*a*}:*x* ∈ Ω and Ω − *a* := {*x* − *a* : *x* ∈ Ω }

By using the paradigm (described above in this section), we may construct the following augmented matrix showing basic variable corresponding to each row.

$$\begin{array}{cc} & \begin{array}{cccccc} \;x_{N}\; & \;x_{L}^{+}\; & \;x_{L\;}^{-} & x_{L^{\prime }}^{+}\; & & b\; \end{array}\\ \begin{array}{c} w\\ x_{L}^{-}\\ x_{L^{\prime }}^{+} \end{array} & \left[\begin{array}{cccccc} \sum_{p+i\in L}a_{iN} & 1 & \;0\; & \;0 & & \sum_{p+i\in L}b_{i}\\ a_{\left(L-p\right)\times N} & I & \;-I & \;0 & & b_{\left(L-p\right)}\\ a_{\left(L^{\prime }-p\right)\times N} & 0 & \;0 & \;I & & b_{\left(L^{\prime }-p\right)} \end{array}\right] \end{array}$$

Here top row (*w*-row) represents infeasibility objective function.

Constructing a short table of the above matrix,

$$\begin{array}{cc} & \begin{array}{ccc} \;b\; & \;x_{N}\; & \;x_{L}^{+}\; \end{array}\\ \begin{array}{c} w\\ -x_{L}^{-}\\ x_{L^{\prime }}^{+} \end{array} & \left[\begin{array}{ccc} \sum_{p+i\in L}b_{i} & \sum_{p+i\in L}a_{iN} & 1\\ b_{\left(L-p\right)} & a_{\left(L-p\right)\times N} & I\\ b_{\left(L^{\prime }-p\right)} & a_{\left(L^{\prime }-p\right)\times N} & 0 \end{array}\right] \end{array}$$

where, ***b***_*L*−*p*_ < 0 and ***b***_*L*’-*p*_ ≥ 0. The procedure to find the entering basic variable is similar to phase 1 simplex method i.e. we may identify the entering basic variable by seeking most negative element in *w*-row (excluding first element, which is actually equal to sum of all infeasibilities). After determining the entering basic variable leaving variable is determined by taking the minimum-ratio test. But the criterion for minimum ratio test would be consequently different for the rows of positive basic variables $x_{L^{\prime }}^{+}$ and negative basic variables $-x_{L}^{-}$.

### New form of Minimum ratio test

Here ratio test scheme is different for positive ‘$x_{L^{\prime }}^{+}$ ‘ and negative ‘$-x_{L}^{-}$ ‘ variables. Ratios corresponding to variables in $x_{L^{\prime }}^{+}$ are obtainable by dividing **b**_*L*’-*p*_ by its corresponding element in the pivot column (only if corresponding element in pivot column is positive) whereas; ratios corresponding to variables in $-x_{L}^{-}$ are obtainable by dividing **b**_*L*−*p*_ by its corresponding element in the pivot column (only if corresponding element in pivot column is negative). The element in the pivot column corresponding to the minimum ratio would be the pivot element. Hence the leaving basic variable would be the variable corresponding to that pivot element.

If the leaving basic variable, say $x_{i}^{-}$,belongs to $x_{L}^{-}$ then set *L* := *L*\{*i*} for the next pivoting table and perform the pivot operation. Now in the new pivoting table proceed with the same procedure of entering and leaving basic variable until *L* becomes empty.

### Reduced short table

The short table size can be more reduced by considering the fact that column of $x_{L}^{+}$ is conjugate of the hidden basic column of $-x_{L}^{-}$ except the coefficient of $x_{L}^{+}$ in infeasibility objective is ‘1’ rather than ‘0’.

One can observe for *i* ∈ *L*, after the pivot operation, columns of $-x_{i}^{-}$ and $x_{i}^{+}$ are identical with the exception that the infeasibility objective coefficient of $x_{i}^{+}$ is greater than infeasibility objective coefficient of $-x_{i}^{-}$ by ‘1’, that is *w*_*i*_ := *w*_*i*_ + 1. So, we can also hide the column of $x_{L}^{+}$ keeping in mind that for every leaving basic variable $x_{i}^{-}\in x_{L}^{-}$ after the pivot operation we must replace the newly obtained non-basic column of $-x_{i}^{-}$ by $x_{i}^{+}$.

$$\begin{array}{cc} & \begin{array}{ccc} b\; & \;x_{N}\; & \end{array}\\ \begin{array}{c} w\\ -x_{L}^{-}\\ x_{L'}^{+} \end{array} & \left[\begin{array}{ccc} \sum_{p+i\in L}b_{i} & \sum_{p+i\in L}a_{iN} & \\ b_{\left(L-p\right)} & a_{\left(L-p\right)\times N} & \\ b_{\left(L'-p\right)} & a_{\left(L'-p\right)\times N} & \end{array}\right] \end{array}$$

Let the elements of reduced short table are denoted by *d*_*ij*_, where *i* ∈ *B* ⋃ {0} and *j* ∈ *N* ⋃ {0},

$$\begin{array}{cc} & \begin{array}{ccc} b & \;x_{N} & \end{array}\\ \begin{array}{c} w\\ -x_{L}^{-}\\ x_{L^{\prime }}^{+} \end{array} & \left[\begin{array}{ccc} \sum_{p+i\in L}d_{i0} & \sum_{p+i\in L}d_{iN} & \\ d_{L0} & d_{LN} & \\ d_{L^{\prime }0} & d_{L^{\prime }N} & \end{array}\right] \end{array}$$

**Note:** Recall that $x_{i}^{}=x_{i}^{+}-x_{i}^{-}$, it is clear that if leaving basic variable is $-x_{i}^{-}$, then $-x_{i}^{-}$ could be replaced by *x*_*i*_ after pivot in the short table.

#### Problem 1

Given a reduced short table *D*(*B*), obtain primal feasibility.

#### Algorithm: Art-free Simplex Method (ASM)

- **Step 1:** Let *L* be a maximal subset of *B* such that *L* = {*i*:*d*_*i*0_ < 0, *i* ∈ *B*}. If *L* = *φ* then *D*(*B*) is primal feasible. **Exit.**
- **Step 2:** Denote the basic variables **x**_*L*_ by $-x_{L}^{-}$ and compute infeasibility objective vector $w\left(B\right)\in {ℜ}^{N}$ such that *w*_*j*_ = Σ_*i*∈*L*_ *d*_*ij*_, *j* ∈ *N*. Place *w* to the top of the reduced short table *D*(*B*).
- **Step 3:** Let *K* ⊆ *N* such that *K* = {*j*: *w*_*j*_ < 0, *j* ∈ *N*}. If *K* = *φ* then *D*(*B*) is primal inconsistent. **Exit.**
- **Step 4:** Choose *k* ∈ *K* such that *w*_*k*_ ≤ *w*_*h*_, ∀*h* ∈ *K*
  (Ties are broken arbitrarily)
- **Step 5:** Choose *r*_1_ ∈ *L* and *r*_2_ ∈ *B* \*L* such that
  $$r_{1}=\arg\min\left\{\left\{\frac{d_{i0}}{d_{ik}}\;|\;\left(d_{i0}\leq 0,d_{ik}<0\right)\right\},i\in L\right\}r_{2}=\arg\min\left\{\left\{\frac{d_{i0}}{d_{ik}}\;|\;\left(d_{i0}\geq 0,d_{ik}>0\right)\right\},i\in B\backslash L\right\}$$
  Set $r:=\arg\min\left\{\frac{d_{r_{1}0}}{d_{r_{1}k}},\frac{d_{r_{2}0}}{d_{r_{2}k}}\right\}$
- **Step 6:** Make a pivot on (*r*, *k*) (⇒ Set *B* := (*B* ⋃ {*k*})\{*r*}, *N* := (*N* ⋃ {*r*})\{*k*} and update *D*(*B*) along with the appended *w*(*B*)).
- **Step 7:** If *r* ∈ *L*, set *L* := *L*\{*r*} and *w*_*r*_ := *w*_*r*_ + 1, replace notation of $-x_{r}^{-}$ by *x*_*r*_
- **Step 8:** If *L* = *φ* then *D*(*B*) is primal feasible. **Exit.**
  Otherwise, go to Step 3.

#### Proof of Correctness

As stated earlier, the objective of the simplex phase 1 is, to minimize the sum of all artificial variables. In an analogous sense, as shown in the last section the infeasibility objective *w* is to $\text{Minimize}\sum_{p+i\in L}x_{p+i}^{-}$. Just like simplex method, our method’s ratio test, by taking minimum of all ratios, preserves the feasibility of existing feasible variables. Hence in the streamlined art-free simplex method the number of negative basic variables $-x_{L}^{-}$ is steadily decreasing. The overall structure of pivoting, forces $\sum_{p+i\in L}b_{i}$ to be strictly increasing for non-degenerate pivots throughout the iterations and constant for degenerate pivots. So, finiteness of total number of bases in every LP problem proves finiteness of our method for complete non-degenerate LP problems.

#### Example 1

Obtain a feasible basis of the following system of inequalities using ASM.

$$\begin{array}{l} -8x_{1}+\;x_{2}-5x_{3}\;\geq 6\\ \;8x_{1}+8x_{2}\geq 8\\ \;-7x_{1}+9x_{2}\geq 1\\ -7x_{1}+7x_{2}-9x_{3}\geq 6\\ x_{1}\geq 0,\;x_{2}\geq 0,\;x_{3}\geq 0 \end{array}$$

By adding non-negative slack variables *x*_4_, *x*_5_, *x*_6_, and *x*_7_ we can construct the associated reduced short table along with row vector *w* (sum of rows of infeasible basic variables) of the above problem as

Initial table:

$$\begin{array}{cc} & \begin{array}{cccc} \;b & \;x_{1} & \;x_{2} & x_{3} \end{array}\\ \begin{array}{c} w\\ x_{4}\\ x_{5}\\ x_{6}\\ x_{7} \end{array} & \left[\begin{array}{cccc} -21 & 14 & -25 & 14\\ -6 & 8 & -1 & 5\\ -8 & -8 & -8 & 0\\ -1 & 7 & -9 & 0\\ -6 & 7 & -7 & 9 \end{array}\right] \end{array}$$

Clearly, current basic solution is infeasible.

Here *L* = {4,5,6,7}, replace $x_{L}^{}\to -x_{L}^{-}$

$$\begin{array}{cc} & \begin{array}{cccc} \;b & \;x_{1} & \;x_{2} & x_{3} \end{array}\\ \begin{array}{c} w\\ -x_{4}^{-}\\ -x_{5}^{-}\\ -x_{6}^{-}\\ -x_{7}^{-} \end{array} & \left[\begin{array}{cccc} -21 & 14 & -25 & 14\\ -6 & 8 & -1 & 5\\ -8 & -8 & -8 & 0\\ -1 & 7 & -9* & 0\\ -6 & 7 & -7 & 9 \end{array}\right] \end{array}$$

Here *B* = {4,5,6,7} and *N* = {1,2,3}, According to most negative coefficient rule *k* = 2, so entering basic variable is *x*_2_ and according to new minimum ratio test *r* = 6 the leaving basic variable is ‘$-x_{6}^{-}$ ‘. Perform the pivot operation on (6,2).

Replace $-x_{6}^{-}\to x_{6}^{}$, *L* = {4,5,6,7}\{6} = {4,5,7}, *w*_6_ := *w*_6_ + 1

$$\begin{array}{cc} & \begin{array}{cccc} \;b & \;x_{1} & \;x_{6} & \;x_{3} \end{array}\\ \begin{array}{c} w\\ -x_{4}^{-}\\ -x_{5}^{-}\\ x_{2}\\ -x_{7}^{-} \end{array} & \left[\begin{array}{cccc} -164/9 & -49/9 & -16/9 & 14\\ -53/9 & 65/9 & -1/9 & 5\\ -64/9 & -128/9* & -8/9 & 0\\ 1/9 & -7/9 & -1/9 & 0\\ -47/9 & 14/9 & -7/9 & 9 \end{array}\right] \end{array}$$

It can be seen that now a single infeasibility is removed.

Iteration 2:

Here, *k* = 1 and *r* = 5 perform pivot operation on (5,1).

Since *r* ∈ *L*, replace $-x_{5}^{-}\to x_{5}^{},$ *L* = {4,5,7}\{5} = {4,7}, *w*_5_ := *w*_5_ + 1

$$\begin{array}{cc} & \begin{array}{cccc} \;b & \;x_{5} & \;x_{6} & \;x_{3} \end{array}\\ \begin{array}{c} w\\ -x_{4}^{-}\\ x_{1}\\ x_{2}\\ -x_{7}^{-} \end{array} & \left[\begin{array}{cccc} -31/2 & 79/128 & -23/16 & 14\\ -19/2 & 65/128 & -9/16 & 5\\ 1/2 & -9/128 & 1/16 & 0\\ 1/2 & -7/128 & -1/16 & 0\\ -6 & 7/64 & -7/8* & 9 \end{array}\right] \end{array}$$

Iteration 3:

Here, *k* = 6 and *r* = 7 perform pivot operation on (7,6).

Since *r* ∈ *L*, replace $-x_{7}^{-}\to x_{7}^{},$ *L* = {4,7}\{7} = {4}, *w*_7_ := *w*_7_ + 1

$$\begin{array}{cc} & \begin{array}{cccc} \;b & \;x_{5} & \;x_{7} & \;x_{3} \end{array}\\ \begin{array}{c} w\\ -x_{4}^{-}\\ x_{1}\\ x_{2}\\ x_{6} \end{array} & \left[\begin{array}{cccc} -79/14 & 7/16 & -9/14 & -11/14\\ -79/14 & 7/16 & -9/14 & -11/14\\ 1/14 & -1/16 & 1/14 & 9/14*\\ 13/14 & -1/16 & -1/14 & -9/14\\ 48/7 & -1/8 & -8/7 & -72/7 \end{array}\right] \end{array}$$

Iteration 4:

Here, *k* = 3 and *r* = 1 perform pivot operation on (1,3).

$$\begin{array}{cc} & \begin{array}{cccc} \;b & \;x_{5} & \;x_{7} & \;x_{1} \end{array}\\ \begin{array}{c} w\\ -x_{4}^{-}\\ x_{3}\\ x_{2}\\ x_{6} \end{array} & \left[\begin{array}{cccc} -50/9 & 13/36 & -5/9 & 11/9\\ -50/9 & 13/36 & -5/9 & 11/9\\ 1/9 & -7/72 & 1/9* & 14/9\\ 1 & -1/8 & 0 & 1\\ 8 & -9/8 & 0 & 16 \end{array}\right] \end{array}$$

Iteration 5:

Here, *k* = 7 and *r* = 3 perform pivot operation on (3,7).

$$\begin{array}{cc} & \begin{array}{cccc} \;b & \;x_{5} & x_{3} & x_{1} \end{array}\\ \begin{array}{c} w\\ -x_{4}^{-}\\ x_{7}\\ x_{2}\\ x_{6} \end{array} & \left[\begin{array}{cccc} -5 & -1/8 & 5 & 9\\ -5 & -1/8* & 5 & 9\\ 1 & -7/8 & 9 & 14\\ 1 & -1/8 & 0 & 1\\ 8 & -9/8 & 0 & 16 \end{array}\right] \end{array}$$

Iteration 6:

Here, *k* = 5 and *r* = 4 perform pivot operation on(4,5).

Since *r* ∈ *L*, replace $-x_{4}^{-}\to x_{4}$, *L* = {4}\{4} = *φ*, *w*_4_ := *w*_4_ + 1

$$\begin{array}{cc} & \begin{array}{cccc} \;b & \;x_{4}\; & x_{3}\; & x_{1} \end{array}\\ \begin{array}{c} w\\ x_{5}\\ x_{7}\\ x_{2}\\ x_{6} \end{array} & \left[\begin{array}{cccc} 0 & 0 & 0 & 0\\ 40 & -8 & -40 & -72\\ 36 & -7 & -26 & -49\\ 6 & -1 & -5 & -8\\ 53 & -9 & -45 & -65 \end{array}\right] \end{array}$$

Now all the infeasibilities have been removed. Primal feasibility is achieved; the feasible solution is (*x*_1_, *x*_2_, *x*_3_) = (0,6,0).

**Fig 4:**
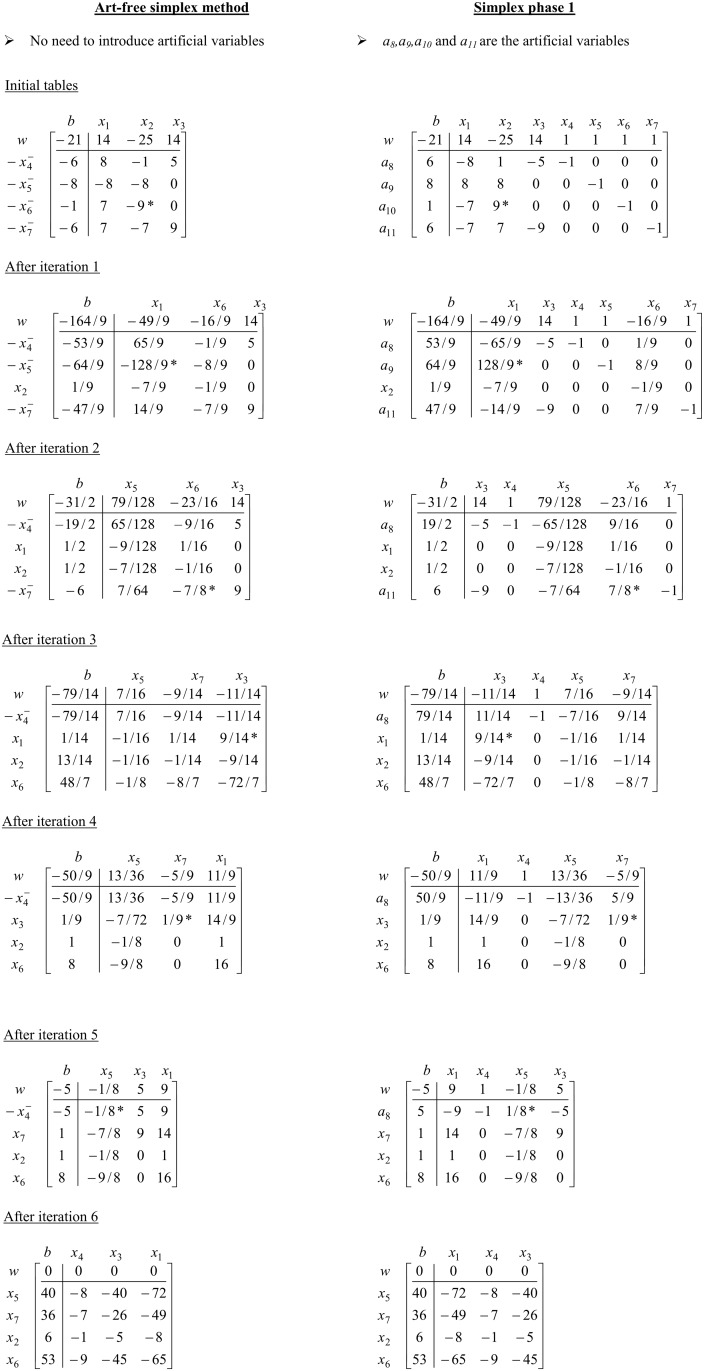
Iteration by iteration comparison between ASM and Simplex method for example 1.

For more illustration purpose see Fig. 4 for an iteration by iteration comparison between Art-free simplex (ASM) and simplex method (SM) phase 1 of the above example.

Remark:

The number of iterations and the pivoting sequence of art-free simplex and simplex phase 1 are same, but it could be easily seen that number of computations are noticeably reduced. For more details, see the computational results section.

#### Example 2

Show that the following system of inequalities is inconsistent by using Art-free simplex method and usual simplex method.

$$\begin{array}{l} 2x_{1}+\;x_{2}\geq 10\\ \;x_{1}+x_{2}\;\leq 1\\ 3x_{1}-x_{2}\geq 2\\ x_{1}\geq 0,\;x_{2}\geq 0 \end{array}$$

Initial table

$$\begin{array}{cc} & \begin{array}{ccc} \;b & \text{​​​ }x_{1} & \;x_{2} \end{array}\\ \begin{array}{c} w\\ -x_{3}^{-}\\ x_{4}\\ -x_{5}^{-} \end{array} & \left[\begin{array}{ccc} -12 & -5 & 0\\ -10 & -2 & -1\\ 1 & 1 & 1\\ -2 & -3* & 1 \end{array}\right] \end{array}$$

Iteration 1

$$\begin{array}{cc} & \begin{array}{ccc} \;b & \text{​​​ }x_{5} & \;x_{2} \end{array}\\ \begin{array}{c} w\\ -x_{3}^{-}\\ x_{4}\\ x_{1} \end{array} & \left[\begin{array}{ccc} -26/3 & -2/3 & -5/3\\ -26/3 & -2/3 & -5/3\\ 1/3 & 1/3 & 4/3*\\ 2/3 & -1/3 & -1/3 \end{array}\right] \end{array}$$

Iteration 2

$$\begin{array}{cc} & \begin{array}{ccc} \;b & \text{​​​ }x_{5} & \;x_{4} \end{array}\\ \begin{array}{c} w\\ -x_{3}^{-}\\ x_{2}\\ x_{1} \end{array} & \left[\begin{array}{ccc} -33/4 & -1/4 & 5/4\\ -33/4 & -1/4 & 5/4\\ 1/4 & 1/4* & 3/4\\ 3/4 & -1/4 & 1/4 \end{array}\right] \end{array}$$

I

Iteration 3

$$\begin{array}{cc} & \begin{array}{ccc} \;b & \text{​}x_{2} & x_{4} \end{array}\\ \begin{array}{c} w\\ -x_{3}^{-}\\ x_{5}\\ x_{1} \end{array} & \left[\begin{array}{ccc} -8 & 1 & 2\\ -8 & 1 & 2\\ 1 & 4 & 3\\ 1 & 1 & 1 \end{array}\right] \end{array}$$

**Fig 5:**
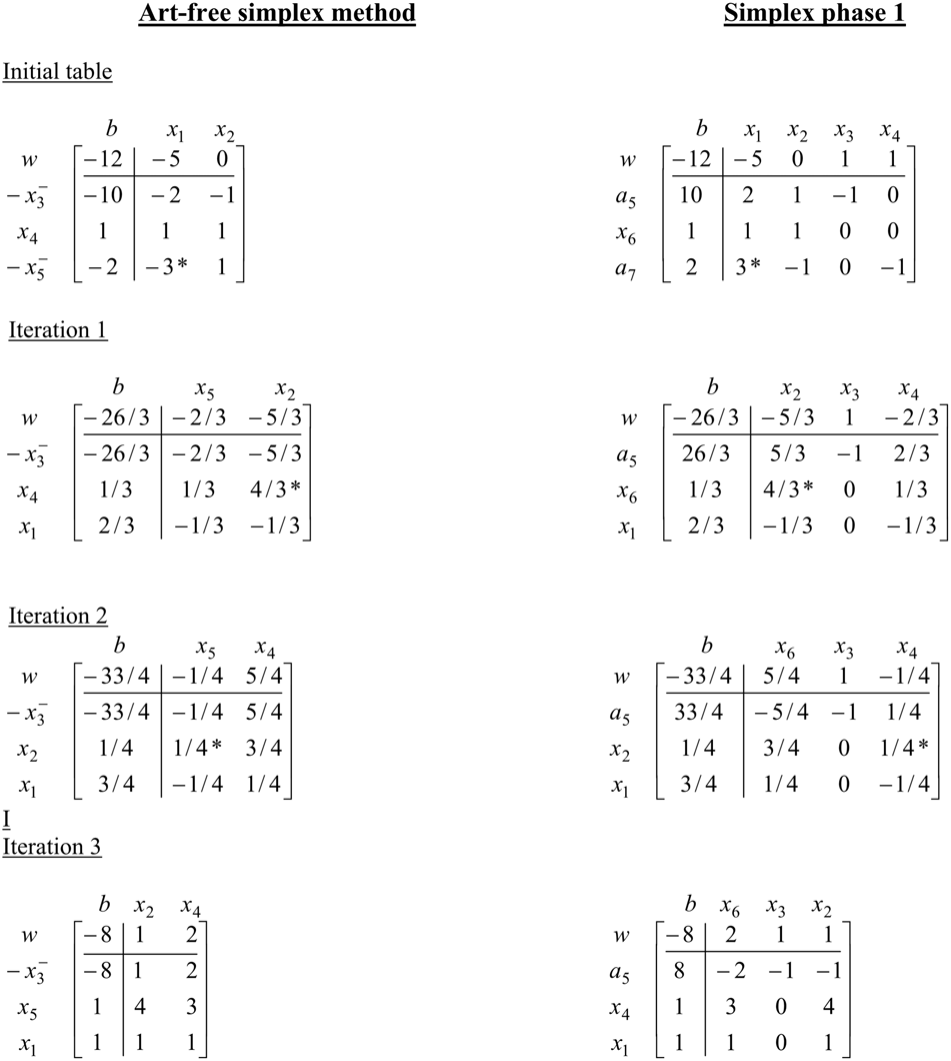
Iteration by iteration comparison between ASM and Simplex method for example 2.

The above table shows that the algorithm terminated unsuccessfully. Hence the problem is primal inconsistent. See Fig. 5 for comparison with simplex method.

## Dual Version of Art-Free Simplex Method

The symmetric relationship between primal dual linear programs always enables one to construct a dual version of every method developed for solving the primal problem. The most popular example of such beautiful characteristic is the dual simplex method. Dual simplex method is known for its application in sensitivity analysis, integer programming, branch-and-bound/cut/price algorithms. But unfortunately phase 1 of dual simplex has not much participated in to the practice of solving dual LPs [24].

In phase 1 of dual simplex, to obtain a dual feasible basis of a given LP problem an additional dual slack variable is introduced which is then assigned an adequately large cost coefficient *M* in the dual objective function. This is analogous to adding a constraint to the primal problem. The method also requires the addition of artificial variables to transform the LP into the standard form. However this approach becomes tedious as the size of the problem is considerably increased. Another reason for not using this approach in practice is that it depends on the value of *M*, a high value might result in numerical problems whereas a value too small might fail to produce a primal feasible solution [24].

In this section we are going to develop dual version of the Art-free simplex method, in fact development of this pivot algorithm is a trivial task. Without going into the geometric or algebraic complications we may construct a dual version of the method by keeping in mind that the aim is to solve the dual feasibility problem through primal short table. Consider the following LP problem,

$$\begin{array}{cc} \text{Maximize} & 5x_{1}-2\;x_{2}+7x_{3}\\ \text{subject to} & \\ & \begin{array}{l} -8x_{1}+\;x_{2}-5x_{3}\;\geq 6\\ \;8x_{1}+8x_{2}\geq 8\\ \;-7x_{1}+9x_{2}\geq 1\\ -7x_{1}+7x_{2}-9x_{3}\geq 6\\ x_{1}\geq 0,\;x_{2}\geq 0,\;x_{3}\geq 0 \end{array} \end{array}$$ (10)

By adding non-negative slack variables *x*_4_, *x*_5_, *x*_6_ and *x*_7_, the associated short table of the above problem can be constructed as shown below. Here, the dual variables *y*_1_, *y*_2_, *y*_3_, *y*_4_, *y*_5,_ *y*_6_ and *y*_7_ have been demonstrated explicitly as it is required to observe dual variables too.

$$\begin{array}{ccc} & \begin{array}{cccc} \;b & \;x_{1} & \;x_{2} & \;x_{3} \end{array} & \\ \begin{array}{c} z\\ x_{4}\\ x_{5}\\ x_{6}\\ x_{7} \end{array} & \left[\begin{array}{cccc} 0 & -5 & 2 & -7\\ -6 & 8 & -1 & 5\\ -8 & -8 & -8 & 0\\ -1 & 7 & -9 & 0\\ -6 & 7 & -7 & 9 \end{array}\right] & \begin{array}{c} y_{4}\\ y_{5}\\ y_{6}\\ y_{7} \end{array}\\ & \begin{array}{cccc} -y & y_{1} & \;y_{2} & y_{3} \end{array} & \end{array}$$

Here *y* is the dual objective variable. Objective coefficients (z-coefficients) of primal non-basic variables are the values of dual basic variables, and values of primal basic variables are coefficients of dual non-basic variables in dual objective.

That is, primal and dual solutions associated with above short table are,

$$x_{1}=0,\;x_{2}=0,\;x_{3}=0,\;x_{4}=-6,\;x_{5}=-8,\;x_{6}=-1,\;x_{7}=-6y_{1}=-5,\;y_{2}=2,\;y_{3}=-7,\;y_{4}=0,\;y_{5}=0,\;y_{6}=0,\;y_{7}=0$$

With mutual objective values z = − y = 0

Clearly, current primal and dual both basic solution are infeasible.

**Fig 6:**
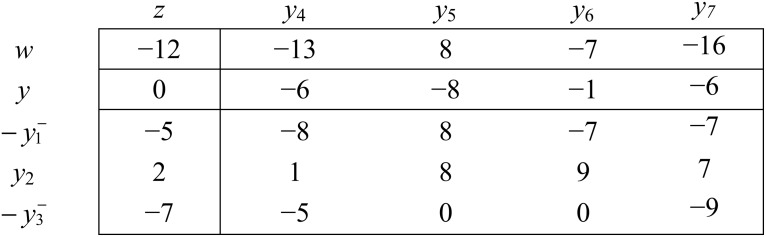
Reduced dual short table for system (10) along with the infeasibility objective w.

**Fig 7:**
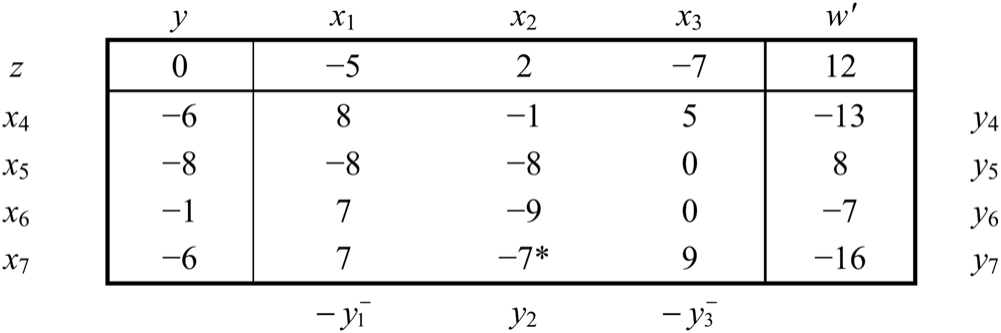
Reduced primal short table for system (10) along with the dual infeasibility objective w’.

As demonstrated earlier in this manuscript to achieve optimality one may either have to achieve primal feasibility and then go for optimality through primal simplex method, or on the other hand one may have to achieve dual feasibility and then go for optimality through dual simplex method. If we measure the degree of infeasibility through the number of infeasible variables, one can see that the above problem is more primal infeasible than dual infeasible. So, from a general perspective the primal feasibility achievement problem might be a difficult task in comparing to dual feasibility achievement problem. For system (10), to attain dual feasibility we may either consider solving it by using art-free simplex method on its reduced dual short table see Fig. 6 or using dual art-free simplex on its reduced primal short table see Fig. 7.

### Problem 2

Given a short table *D*(*B*), obtain dual feasibility.

### Algorithm: Dual Art-free Simplex Method (DASM)

- **Step 1:** Let *K* be a maximal subset of *B* such that *K* = {*j* : *d*_0*j*_ < 0, *j* ∈ *N*}. If *K* = *φ* then *D*(*B*) is dual feasible. **Exit.**
- **Step 2:** Denote the basic variables **y**_*K*_ by $-y_{K}^{-}$ and compute dual infeasibility objective vector $w^{\prime }\left(B\right)\in {ℜ}^{B}$ such that ${w^{\prime }}_{i}=\sum_{j\in K}d_{ij},\;i\in B$. Append *w*’ to the right of the dictionary *D*(*B*).
- **Step 3:** Let *L* ⊆ *B* such that $L=\left\{i:{w^{\prime }}_{i}<0,i\in B\right\}$. If *L* = *φ* then *D*(*B*) is dual inconsistent. **Exit.**
- **Step 4:** Choose *r* ∈ *L* such that ${w^{\prime }}_{r}\leq {w^{\prime }}_{l}\;\forall l\in L$
  (Ties are broken arbitrarily)
- **Step 5:** Choose *k*_1_ ∈ *K* and *k*_2_ ∈ *N*\*K* such that
  $$\begin{array}{l} k_{1}=\arg\max\left\{\left\{\frac{d_{0j}}{d_{rj}}\;|\;\left(d_{0j}\leq 0,d_{rj}>0\right)\right\},j\in K\right\}\\ k_{2}=\arg\max\left\{\left\{\frac{d_{0j}}{d_{rj}}\;|\;\left(d_{0j}\geq 0,d_{rj}<0\right)\right\},j\in N\backslash K\right\} \end{array}$$
  Set $k:=\arg\max\left\{\frac{d_{0k_{1}}}{d_{rk_{1}}},\frac{d_{0k_{2}}}{d_{rk_{2}}}\right\}$
- **Step 6:** Make a pivot on (*r*, *k*) (⇒ Set *B* := (*B* ⋃ {*k*})\{*r*}, *N* := (*N* ⋃ {*r*})\{*k*} and update *D*(*B*) along with the appended *w*’(*B*)).
- **Step 7:** If *k* ∈ *K*, *K* := *K*\{*k*} and ${w^{\prime }}_{k}:={w^{\prime }}_{k}+1$, replace notation of $-y_{k}^{-}$ by **y**_*k*_
- **Step 8:** If *K* = *φ* then *D*(*B*) is dual feasible. **Exit.**
  Otherwise, go to Step 3.

### Example 3

Obtain a complementary dual feasible basis for the associated LP of the following short table by using Dual Art-free simplex method.

$$\begin{array}{ccc} & \begin{array}{cccc} \;b & \;x_{1} & \;x_{2} & \;x_{3} \end{array} & \\ \begin{array}{c} z\\ x_{4}\\ x_{5}\\ x_{6}\\ x_{7} \end{array} & \left[\begin{array}{cccc} 0 & -5 & 2 & -7\\ -6 & 8 & -1 & 5\\ -8 & -8 & -8 & 0\\ -1 & 7 & -9 & 0\\ -6 & 7 & -7 & 9 \end{array}\right] & \begin{array}{c} y_{4}\\ y_{5}\\ y_{6}\\ y_{7} \end{array}\\ & \begin{array}{cccc} & \;y_{1} & \;y_{2} & y_{3} \end{array} & \end{array}$$

Here *K* = {1,3}, replace $y_{K}^{}\to -y_{K}^{-}$

b    x1 x2    x3 w​ 'zx4x5x6x7[0−52−712−68−15−13−8−8−808−17−90−7−67−7*9−16]y4y5y6y7−y1−y2−y3−

Iteration 1:

Here, *B* = {4,5,6,7} and *N* = {1,2,3}, According to most negative dual coefficient rule *k* = 2, so leaving dual basic variable is y_2_ and according to art-free dual minimum ratio test *r* = 7 the entering dual basic variable is ‘y_7_’. Perform the pivot operation on (7,2)

    b         x1     x7      x3           w​ 'zx4x5x6x2[−12/7−32/7−31/752/7−36/77*−1/726/7−75/7−8/7−16−8/7−72/7184/747/7−2−9/7−81/795/76/7−1−1/7−9/716/7]y4y5y6y2−y1−      y7      −y3−

Iteration 2:

Here, *k* = 1 and *r* = 4 perform pivot operation on (4,1).

Since *k* = 1 ∈ *k*, replace $-y_{1}^{-}\to y_{1}^{},\;K=\left\{1,3\right\}\backslash \left\{1\right\}=\left\{3\right\},\;{w^{\prime }}_{1}:={w^{\prime }}_{1}+1$

$$\begin{array}{l} \begin{array}{ccc} & \;\begin{array}{ccccc} b & \;x_{4} & \;x_{7} & \;x_{3}\; & \text{ ​ }w\;' \end{array} & \\ \begin{array}{c} z\\ x_{1}\\ x_{5}\\ x_{6}\\ x_{2} \end{array} & \left[\begin{array}{ccccc} -192/49 & 3/7 & 11/49 & -139/49 & 139/49\\ -36/49 & 1/7 & -1/49 & 26/49* & -26/49\\ -632/49 & 16/7 & -72/49 & -88/49 & 88/49\\ 257/49 & 2/7 & -65/49 & -515/49 & 515/49\\ 6/49 & 1/7 & -8/49 & -37/49 & 37/49 \end{array}\right] & \begin{array}{c} y_{1}\\ y_{5}\\ y_{6}\\ y_{2} \end{array}\\ & \begin{array}{ccccc} & y_{4} & \;y_{7} & \;-y_{3}^{-} & \end{array} & \end{array} \end{array}$$

Iteration 3:

Here, *k* = 3 and *r* = 1 perform pivot operation on (1,3).

Since *k* = 3 ∈ *k*, replace $-y_{3}^{-}\to y_{3}^{},\;K=\left\{3\right\}\backslash \left\{3\right\}=\left\{\right\},\;{w^{\prime }}_{3}:={w^{\prime }}_{3}+1$

$$\begin{array}{l} \begin{array}{ccc} & \;\begin{array}{ccccc} b & \;x_{4} & \;x_{7} & \;x_{1}\; & \;w\;' \end{array} & \\ \begin{array}{c} z\\ x_{3}\\ x_{5}\\ x_{6}\\ x_{2} \end{array} & \left[\begin{array}{ccccc} -102/13 & 31/26 & 3/26 & 139/26 & 0\\ -18/13 & 7/26 & -1/26 & 49/26 & 0\\ -200/13 & 36/13 & -20/13 & 44/13 & 0\\ -121/13 & 81/26 & -45/26 & 515/26 & 0\\ -12/13 & 9/26 & -5/26 & 37/26 & 0 \end{array}\right] & \begin{array}{c} y_{3}\\ y_{5}\\ y_{6}\\ y_{2} \end{array}\\ & \begin{array}{ccccc} & \;y_{4} & \;y_{7} & \;y_{1} & \end{array} & \end{array} \end{array}$$

Dual feasibility is achieved; the complementary dual feasible solution is $\left(x_{1},x_{2},x_{3}\right)=\left(0,-\frac{12}{13},-\frac{18}{13}\right)$.

### Example 4

Show that the following problem is dual inconsistent by using dual art-free simplex method.

$$\begin{array}{cc} \text{Maximize} & 3x_{1}+5\;x_{2}+2x_{3}\\ \text{subject to} & \\ & \begin{array}{l} \;x_{1}-5x_{3}\leq \;4\\ \;x_{2}-4x_{3}\geq \;6\\ 3x_{1}+2x_{2}\;-\;x_{3}\;\geq 18\\ \;x_{1}+\;x_{2}-\;2x_{3}\geq \;8\\ 5x_{1}+4x_{2}-20x_{3}\geq 32\\ x_{1}\geq 0,\;x_{2}\geq 0,\;x_{3}\geq 0 \end{array} \end{array}$$

By adding non-negative slack variables *x*_4_, *x*_5_, *x*_6_, *x*_7_ and *x*_8_ we can construct the associated dictionary along with column vector *w'* (negative sum of columns of infeasible dual basic variables) of the above problem.

Initial table:

$$\begin{array}{ccc} & \begin{array}{ccccc} \;b & \;x_{1} & \;x_{2} & \;x_{3} & \;w\;' \end{array} & \\ \begin{array}{c} z\\ x_{4}\\ x_{5}\\ x_{6}\\ x_{7}\\ x_{8} \end{array} & \left[\begin{array}{ccccc} 0 & -3 & -5 & -2 & 10\\ 4 & 1 & 0 & -5 & 4\\ -6 & 0 & -1 & 4 & -3\\ -18 & -3 & -2 & 1 & 4\\ -8 & -1 & -1 & 2 & 0\\ -32 & -5 & -4 & 20 & -11 \end{array}\right] & \begin{array}{c} y_{4}\\ y_{5}\\ y_{6}\\ y_{7}\\ y_{8} \end{array}\\ & \begin{array}{ccccc} & \;y_{1} & \;y_{2} & y_{3} & \end{array} & \end{array}$$

Here *y*_1_, *y*_2_, *y*_3_, *y*_4_, *y*_5,_ *y*_6,_ *y*_7_ and *y*_8_ are dual variables.

Here *K* = {1,2,3}, replace $y_{K}^{}\to -y_{K}^{-}$

$$\begin{array}{ccc} & \begin{array}{ccccc} \;b & \;x_{1} & \;x_{2} & \;x_{3} & \;w\;' \end{array} & \\ \begin{array}{c} z\\ x_{4}\\ x_{5}\\ x_{6}\\ x_{7}\\ x_{8} \end{array} & \left[\begin{array}{ccccc} 0 & -3 & -5 & -2 & 10\\ 4 & 1 & 0 & -5 & 4\\ -6\; & 0 & -1 & 4 & -3\\ -18\; & -3 & \;-2\; & 1 & 4\\ -8 & -1 & -1 & 2 & 0\\ -32 & -5 & -4 & 20* & -11 \end{array}\right] & \begin{array}{c} y_{4}\\ y_{5}\\ y_{6}\\ y_{7}\\ y_{8} \end{array}\\ & \begin{array}{ccccc} & \;-y_{1}^{-} & -y_{2}^{-} & \;-y_{3}^{-} & \end{array} & \end{array}$$

Iteration 1:

Here, *k* = 3 and *r* = 8 perform pivot operation on (8,3).

Since *k* = 3 ∈ *k*, replace $-y_{3}^{-}\to y_{3}^{},\;K=\left\{1,2,3\right\}\backslash \left\{3\right\}=\left\{1,2\right\},\;{w^{\prime }}_{3}:={w^{\prime }}_{3}+1$

$$\begin{array}{ccc} & \begin{array}{ccccc} \;b & \;x_{1} & \;x_{2} & \;x_{8} & \;w\;' \end{array} & \\ \begin{array}{c} z\\ x_{4}\\ x_{5}\\ x_{6}\\ x_{7}\\ x_{3} \end{array} & \left[\begin{array}{ccccc} -16/5 & -7/2 & -27/5 & 1/10 & 89/10\\ -4 & -1/4 & -1 & 1/4 & 5/4\\ 2/5\; & 1 & -1/5 & -1/5* & -4/5\\ -82/5\; & -11/4 & -9/5 & -1/20 & 91/20\\ -24/5 & -1/2 & -3/5 & -1/10 & 11/10\\ -8/5 & -1/4 & -1/5 & 1/20 & 9/20 \end{array}\right] & \begin{array}{c} y_{4}\\ y_{5}\\ y_{6}\\ y_{7}\\ y_{3} \end{array}\\ & \begin{array}{ccccc} & \;-y_{1}^{-} & \;-y_{2}^{-} & \;y_{8} & \end{array} & \end{array}\;$$

Iteration 2:

Here, *k* = 8 and *r* = 5 perform pivot operation on (5,8)

$$\begin{array}{ccc} & \begin{array}{ccccc} \;b & \;x_{1} & \;x_{2} & \;x_{5} & \;w\;' \end{array} & \\ \begin{array}{c} z\\ x_{4}\\ x_{8}\\ x_{6}\\ x_{7}\\ x_{3} \end{array} & \left[\begin{array}{ccccc} -3 & -3 & -11/2 & 1/2 & 17/2\\ -7/2 & 1 & -5/4 & 5/4 & 1/4\\ -2 & -5 & 1 & -5 & 4\\ -33/2\; & -3 & -7/4 & -1/4 & 19/4\\ -5 & -1 & -1/2 & -1/2 & 3/2\\ -3/2 & 0 & -1/4 & 1/4 & 1/4 \end{array}\right] & \begin{array}{c} y_{4}\\ y_{8}\\ y_{6}\\ y_{7}\\ y_{3} \end{array}\\ & \begin{array}{ccccc} & \;-y_{1}^{-} & \;-y_{2}^{-} & \;y_{5} & \end{array} & \end{array}\;$$

The algorithm has terminated unsuccessfully. Here, the last table shows that the problem is dual inconsistent.

## Applications

Since the proposed method ASM is an efficient alternative to the simplex method phase 1 and dual simplex method so it can effectively be incorporated in the solution process of linear programming problems, integer programming problems, sensitivity analysis, parametric programming etc. It can become an essential tool which can directly be employed by researchers in solving various problems of diverse fields like biological sciences and engineering for example: biological sciences [29] [31], medical sciences [28] [30], biochemical sciences [32], mechanical engineering [33] etc.

## Computational Results

In this section, tables 1 and 2 present a comparison of the computational results of our algorithm with the simplex method. Using random models suggested by Kaluzny [26] we generated 250 consistent linear programs and 250 inconsistent linear programs with the coefficients *c*_*j*_, *b*_*i*_ and *a*_*ij*_ chosen randomly from the integer interval [−50,50]. The results of both consistent and inconsistent problems exhibit that our algorithm (ASM) and the simplex method (SM) both take same number of iterations.

**Table 1:** Average number of iterations on random consistent LPs.

Order	ASM/SM
3x3	1.432
3x5	1.76
3x7	1.796
5x5	2.913
5x10	3.191
7x5	4.177
7x10	5.082
10x5	6.117
10x10	8.128
10x20	7.623
10x25	7.628
15x15	14.407
15x20	14.359
20x20	22.167
20x30	20.164
30x30	41.361
40x40	66.660
50x50	102.079
50x70	87.842
50x100	60.9154
70x50	172.832
70x70	187.799
70x100	152.531
80x80	231.531
90x90	287.652
100x100	347.511

**Table 2:** Average number of iterations on random inconsistent LPs.

Order	ASM/SM
3x3	0.96
3x5	1.332
3x7	1.381
5x5	2.533
5x10	3.571
7x5	3.293
7x10	5.001
10x5	4.236
10x10	7.328
10x20	10.076
10x25	11.465
15x15	12.884
15x20	15.643
20x20	20.385
20x30	27.136
30x30	41.398
40x40	66.911
50x50	94.007
50x70	125.203
50x100	136.697
70x50	108.923
70x70	174.128
70x100	230.393
80x80	219.281
90x90	279.052
100x100	317.426

For further testing on practical problems, we executed both the algorithms on NETLIB problems [34] which again revealed that for each problem number of iterations taken by both algorithms are exactly the same. The results have been summarized in table 3.

**Table 3:** Number of iterations on 22 instances of NETLIB test problems.

S. No.	Problem Title	Size	ASM/SM
1	ADLITTLE	56x97	22
2	AFIRO	27x32	6
3	AGG2	516x302	47
4	AGG3	516x302	37
5	BANDM	305 x 472	9277
6	BEACONFD	173x262	89
7	BNL1	643x1175	8486
8	E226	223x282	128
9	FFFFF800	524x854	2001
10	ISRAEL	174x142	8
11	SCAGR25	471x500	2051
12	SCAGR7	129x140	249
13	SCFXM2	660x914	1378
14	SCFXM3	990x1371	2408
15	SCORPION	388x358	398
16	SCSD1	77x760	121
17	SCSD6	147x1350	297
18	SCSD8	397x2750	2784
19	SCTAP2	1090x1880	1910
20	SCTAP3	1480x2480	1923
21	SHARE2B	96x79	120
22	STOCFOR1	117x111	56
21	SHARE2B	96x79	120
22	STOCFOR1	117x111	56

**Fig 8:**
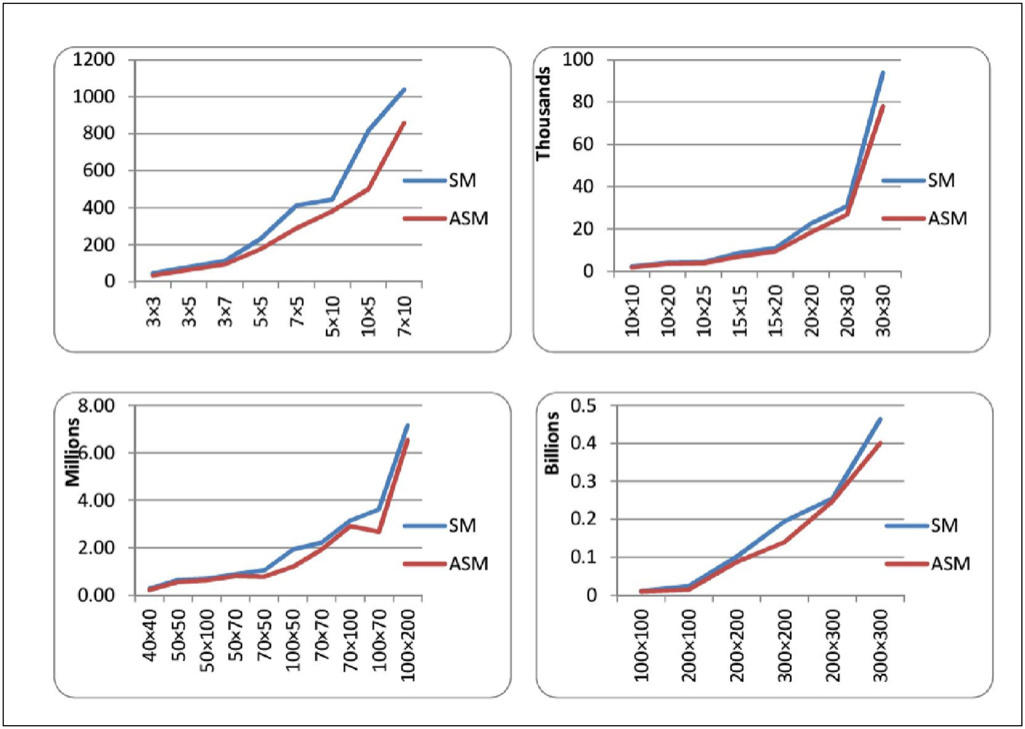
Comparison of SM and ASM in terms of Multiplication Operations.

**Fig 9:**
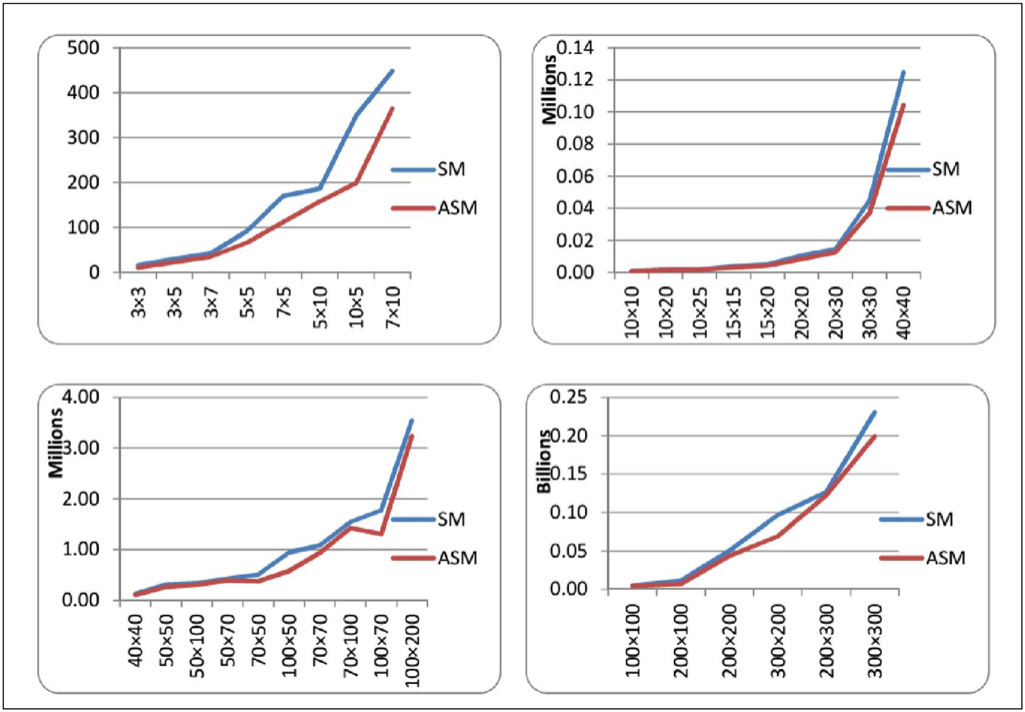
Comparison of SM and ASM in terms of Addition Operations.

**Fig 10:**
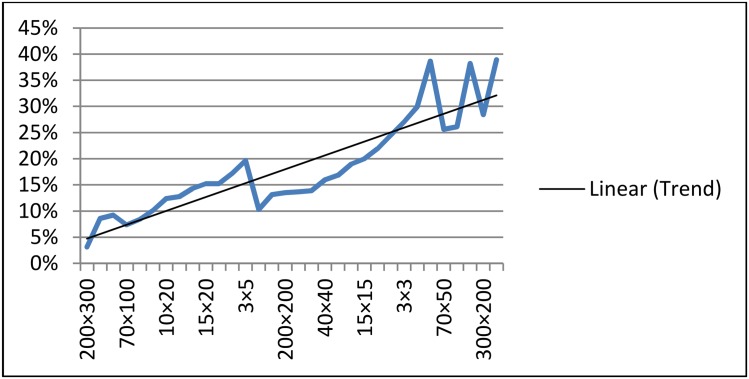
Graph showing trend of percentage of computations saved in ASM.

The above comparisons in between ASM and SM strengthen that ASM and SM always follow exactly same number of iterations. The following tables show a comparison of computational efficiency of both the methods:

Figs. 8 and 9 depict the comparison of SM and ASM by the average number of multiplication and addition operations needed to solve LP problems of different sizes. The tables illustrate that (either we consider multiplications or additions) ASM always need less number of operation computations as compared to SM. This difference becomes quite noticeable especially for the problems that have greater number of constraints as compared to the number of variables. So, it is empirically observed that ASM is much advantageous when “number of constraints minus number of variables” is a large number. This observation could be verified by the graph as depicted in Fig. 10, as the value of *m* − *n* increases the percentage of saved computations in ASM also increases. For example for a 200 × 300 order problem the average saving in computations is just about 5% but in contrast to a 300 × 200 order problem it reaches a remarkable level of 40%. This fact can also be seen in the problems of order 5 × 10, 10 × 5, 50 × 100 and 100 × 70. For the problems having *m* ≈ *n*, the savings in number of computations is not much high. Basic theory of duality asserts that in contrast to ASM, the dual art-free simplex method (DASM) would be more efficient computationally when *n* − *m* is large.

## Conclusion

In this paper streamlined artificial free versions of simplex and dual simplex method have been presented. These new versions do not need any kind of artificial variables or artificial constraints; they could start with any feasible or infeasible basis of an LP, providing full freedom to the user that whether to start with primal artificial free version or dual artificial free version without making any abrupt changes to the LP structure. These methods follow the same pivoting sequence as of simplex phase 1 without showing any explicit description of artificial variables or artificial constraints. Computational results showed that ASM is more efficient when *m* − *n* is large and DASM is more efficient when *n* − *m* is large. Hence these methods also provide great benefits in class room teaching by eliminating the relatively difficult and tedious calculations of artificial variables and constraints. It is also a teaching aid for the teachers who want to teach feasibility achievement as a separate topic before teaching optimality achievement. It is very helpful tool in integer programming and sensitivity analysis, because it provides a way to avoid dual simplex method.
